# Fast Method for Excited-State Dynamics in Complex Systems and Its Application to the Photoactivation of a Blue Light Using Flavin Photoreceptor

**DOI:** 10.1021/acs.jpclett.2c03797

**Published:** 2023-01-30

**Authors:** Patrizia Mazzeo, Shaima Hashem, Filippo Lipparini, Lorenzo Cupellini, Benedetta Mennucci

**Affiliations:** Dipartimento di Chimica e Chimica Industriale, Università di Pisa, Via G. Moruzzi 13, 56124 Pisa, Italy

## Abstract

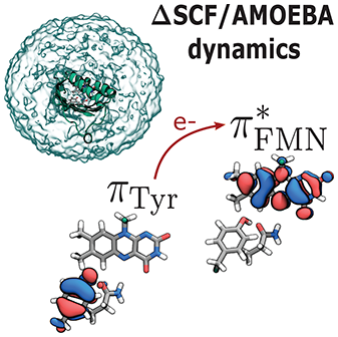

The excited-state dynamics of molecules embedded in complex (bio)matrices is still a challenging goal for quantum chemical models. Hybrid QM/MM models have proven to be an effective strategy, but an optimal combination of accuracy and computational cost still has to be found. Here, we present a method which combines the accuracy of a polarizable embedding QM/MM approach with the computational efficiency of an excited-state self-consistent field method. The newly implemented method is applied to the photoactivation of the blue-light-using flavin (BLUF) domain of the AppA protein. We show that the proton-coupled electron transfer (PCET) process suggested for other BLUF proteins is still valid also for AppA.

In the last few years, the applicability of excited-state (ES) dynamics has significantly increased both in terms of the type of processes that can be described and the complexity of the systems that can be treated. This has been made possible thanks to the combination of quantum mechanical (QM) descriptions with classical models mostly based on molecular mechanics (MM) force fields. Both adiabatic and nonadiabatic formulations of QM/MM ES dynamics have been proposed and successfully applied to a large variety of molecules in an environment of increasing complexity.^1−5^ In these hybrid formulations, the computational cost is mainly determined by the QM method employed. Indeed, QM/MM ES dynamics simulations often exploit relatively fast QM approaches such as time-dependent density functional theory (TD-DFT), but still the computational cost remains high, making it very difficult, if not impossible, to obtain statistically relevant results when large and flexible systems such as biological macromolecules are investigated. An interesting strategy to enhance the applicability of these calculations is to exploit GPU-accelerated routines.^6,7^ However, until now, the most effective speed up has been obtained by introducing semiempirical QM methods in their wave function basis or as DFT formulations as shown by recent studies on photoreceptors and pigment–protein complexes.^8−11^ In both cases, however, a proper parametrization is needed, which largely limits the ease of use and generalizability of the selected semiempirical method. An interesting ab initio alternative is represented by the excited-state self-consistent field methods (also known as ΔSCF).^12−17^ These methodologies are thought to be less computational demanding than TD-DFT while maintaining a high accuracy of the description, especially in the case of conical intersections and charge-transfer (CT) states. ΔSCF proposes to treat the excited state as a single determinant and to optimize molecular orbitals (MOs) at the excited-state level. Notably, ΔSCF solutions can be seen as natural quasidiabatic (from now on, diabatic) states, whose nature does not change upon changing nuclear coordinates.^18^ Thus, ΔSCF is a promising strategy for dealing with ES dynamics that occur on a single diabatic surface, but extensions to nonadiabatic dynamics have been presented as well.^19,20^

Recently we have presented the coupling of ΔSCF methods to a polarizable MM embedding^21^ using the popular AMOEBA force field.^22^ The latter employs fixed charges, dipoles, and quadrupoles to describe the electrostatics and an induced-point-dipole (IPD) model to describe polarization. By performing an extended comparative analysis of the ΔSCF approach and TD-DFT, we have shown that ΔSCF/AMOEBA mainly differs from TD-DFT/AMOEBA as it naturally includes the so-called state-specific (SS) polarization effect. Indeed, ΔSCF/AMOEBA treats the excited state on the same basis as the ground state, including the mutual polarization between excited-state QM density and the environment. This means that the SS response is obtained self-consistently. In TD-DFT/AMOEBA formulations instead an SS effect can be obtained only as a perturbative correction to the energy.^23,24^ On the other hand, ΔSCF methods neglect the other possible response of the polarizable environment, namely, that generated by the transition density that characterizes the excitation, rather than the excited-state density. This response, commonly known as a linear response (LR) but also classified as an excited-state “dispersion” or “resonance” contribution,^24−26^ is the one automatically included in TDDFT/AMOEBA formulations.

The efficient combination of accuracy and the low computational cost of ΔSCF/AMOEBA motivated us to exploit this method for ES dynamics by building on the versatile machinery we have developed in the last few years for (TD)DFT/AMOEBA molecular dynamics simulations.^3,21,27−30^

This machinery uses Tinker^31,32^ as a MD engine and to compute the bonded and van der Waals terms of the energy and forces and then calls a locally modified development version of the Gaussian suite of programs^33^ to compute the QM/AMOEBA energy and forces, which include all electrostatic and polarization contributions. The (TD)DFT/AMOEBA implementation builds on top of a general linear-scaling electrostatic engine^34,35^ based on the fast multipole method which, coupled with an efficient preconditioned conjugate gradient iterative solver^36,37^ for the polarization equations, allows us to treat large and very large MM embeddings at limited computational cost. Furthermore, the coupling between Gaussian and Tinker is efficiently implemented by using the GauOpen^38^ open source library, which allows exchanging data from and to Gaussian in a transparent and efficient way. MD simulations are further accelerated by using advanced extrapolation techniques, such as Niklasson’s extended Lagrangian Born–Oppenheimer method^39,40^ and, more recently, Grassmann extrapolation.^41,42^

The TDDFT/AMOEBA approach suffers, however, from three main limitations. As already commented, a TDDFT energy-force evaluation is costly and the presence of AMOEBA further aggravates this cost. Moreover, TDDFT cannot be used to describe excited states that have marked double excitation character or systems with multireference character such as diradicals. Finally, TDDFT/AMOEBA cannot describe the ES polarization in an SS framework using a self-consistent formulation as would instead be required when, for example, an ES with a large amount of CT character is investigated. Here we show that the ΔSCF method can be a good solution to all of these problems.

ΔSCF describes the ES as a single determinant exactly as the ground state (GS). Starting from GS-optimized MOs, the excited determinant is generated by moving one electron from an occupied to a virtual MO and then optimized through another SCF procedure to obtain an excited-state solution. The one-electron excitation removes the spin symmetry, so ΔSCF iterations are always performed with an open-shell approach, which is normally unrestricted.^12^ To avoid the collapse of the ES wave function onto the GS determinant, the SCF procedure is modified, for example, by changing the way in which new occupied orbitals are selected at each SCF cycle. Different ΔSCF methods adopt various expedients that force the solution to remain in the excited state closest to the initial guess during SCF iterations.^12−17^ Here, we focus on two different strategies, namely, the initial maximum overlap method (iMOM)^13^ and the state-targeted energy projection (STEP).^14^ Both algorithms use the initial guess as a reference set of orbitals. iMOM is a non-Aufbau SCF algorithm that, after obtaining MOs at the *k*th SCF iteration, builds the associated density matrix by choosing the orbitals that have the maximum overlap with the reference, unlike the standard algorithm which chooses the lowest eigenvalues of the Fock (Kohn–Sham) matrix. The STEP algorithm is an Aufbau SCF algorithm where the optimization of the ES is obtained by level shifting the eigenvalues of the orbitals so that the ones corresponding to the reference excited state become the lowest. A more detailed description of these two methods is reported in section S1 of the Supporting Information.

Both ΔSCF strategies have been implemented in a locally modified development version of the Gaussian suite of programs^33^ and coupled in a self-consistent way to the polarizable AMOEBA environment, using the same machinery that is in place for GS simulations, including the extrapolation techniques used to accelerate convergence in MD simulations. The Grassmann extrapolation method^42^ is particularly well suited for ΔSCF MD simulations, as it is transparent to the type of state that has to be extrapolated and depends only on the density matrices of the previous steps and the geometrical parameters. Such a method uses tools from computational differential geometry to perform a linear extrapolation of density matrices while retaining their physical properties and, in particular, idempotency. This is achieved by mapping the density matrices manifold onto its tangent plane, which is a vector space: the linear extrapolation is performed on such a space, and then the extrapolated density is mapped back to the manifold. As the maps between the manifold and its tangent plane are bijective,^41^ no information is lost. The extrapolation coefficients are obtained by fitting a molecular descriptor—we use the Coulomb matrix^42^—at the current step with the ones at the previous *M* steps. In this work, we have generalized such a strategy in two ways.

First, we have extended it to open-shell systems by extrapolating both spin densities (Supporting Information section S2). Second, to preserve the shape and localization of the ES orbitals along the MD simulation and thus to enforce the convergence of the ΔSCF procedure to the state of interest, we use the converged MO coefficients from the previous MD step as reference orbitals for iMOM or to compute the appropriate level shift for STEP. We note here that these coefficients are not consistent with the extrapolated density; however, as these are used only to select the correct state during ΔSCF iterations, this is not a problem in practice.

Overall, the ΔSCF/AMOEBA approach combines the advantages of a naturally state-specific description of the polarization of the environment^21^ with remarkable time saving with respect to a traditional TD-DFT BO-MD simulation: the combination of having a robust guess and avoiding computing the ground state and excited state separately makes ΔSCF/AMOEBA about 6 times faster than TD-DFT/AMOEBA. The newly implemented method is here applied to a very intriguing problem, namely, the photoactivation of a blue light using flavin (BLUF) photoreceptor.

Experimental and computational studies on several BLUF proteins suggest a blue-light-induced mechanism, which passes through a proton-coupled electron transfer (PCET) process.^43−50^ However, for the BLUF domain of the AppA protein from the bacterium *Rhodobacter sphaeroides*, the validity of this mechanism has never been proven. On the contrary, since experiments found no trace of the radical intermediates involved in the PCET process,^51,52^ AppA was classified as a specific case among the BLUF domains. In the literature, alternative mechanisms were proposed^53,54^ but none of them has still found a robust demonstration.

So far, the computational investigation of the photoactivation of AppA has been hindered by the fact that there was not a clear consensus on the identity of both dark and light structures. There are indeed two crystallographic structures that have been resolved for the dark state of AppA, which differ in both the composition of the binding site and the spatial disposition of residues. In both cases, the active site is composed of the flavin, a tyrosine (Tyr21), and a glutamine (Gln63). However, in the structure published in 2005 by Anderson et al.^55^ a tryptophan (Trp104) is present in the binding site (so it was called Trp_*in*_), while in the structure published in 2006 by Jung et al.^56^ a methionine residue (Met106) replaces the Trp104 (so the name is Met_*in*_). The other substantial difference is in the orientation of the Gln63 residue, since in Trp_*in*_ the glutamine–NH_2_ group points toward the Tyr21 and in Met_*in*_ the Gln63 is rotated by 180°, showing a hydrogen bond between Tyr21 and the carbonyl group of glutamine. This ambiguity was recently solved by some of us^57^ by comparing Trp_*in*_ and Met_*in*_ through the combination of molecular dynamics (MD) simulations with calculations of nuclear magnetic resonance (NMR), IR, and UV–vis spectra. The integration of structural and spectroscopic analyses has allowed us to identify the Met_*in*_ as the dark-adapted structure, so from now on, we will refer to this geometry as the dark-adapted state.

Now that we have a clear dark-adapted structure, we can proceed with investigating the photoactivation to verify whether the PCET mechanism identified and proved in other BLUF domains can apply to AppA as well and possibly explain why radical intermediates have not been seen in the experiments. As a starting hypothesis of the photoactivation process, we consider the one first suggested by Domratcheva et al.^45^ and schematically described in Figure 1. According to this hypothesis, one electron is transferred from Tyr21 to the excited flavin, generating a charge-transfer (CT) state which evolves in a proton transfer from tyrosine to flavin, mediated by glutamine (the so-called forward PCET). The resulting imidic acid group (i.e., the glutamine residue after the double proton transfer) rotates, and the system undergoes the so-called reverse PCET: one electron moves from flavin to Tyr21 and a proton transfer from flavin to Tyr21 follows, mediated by the imidic acid.

**Figure 1:**
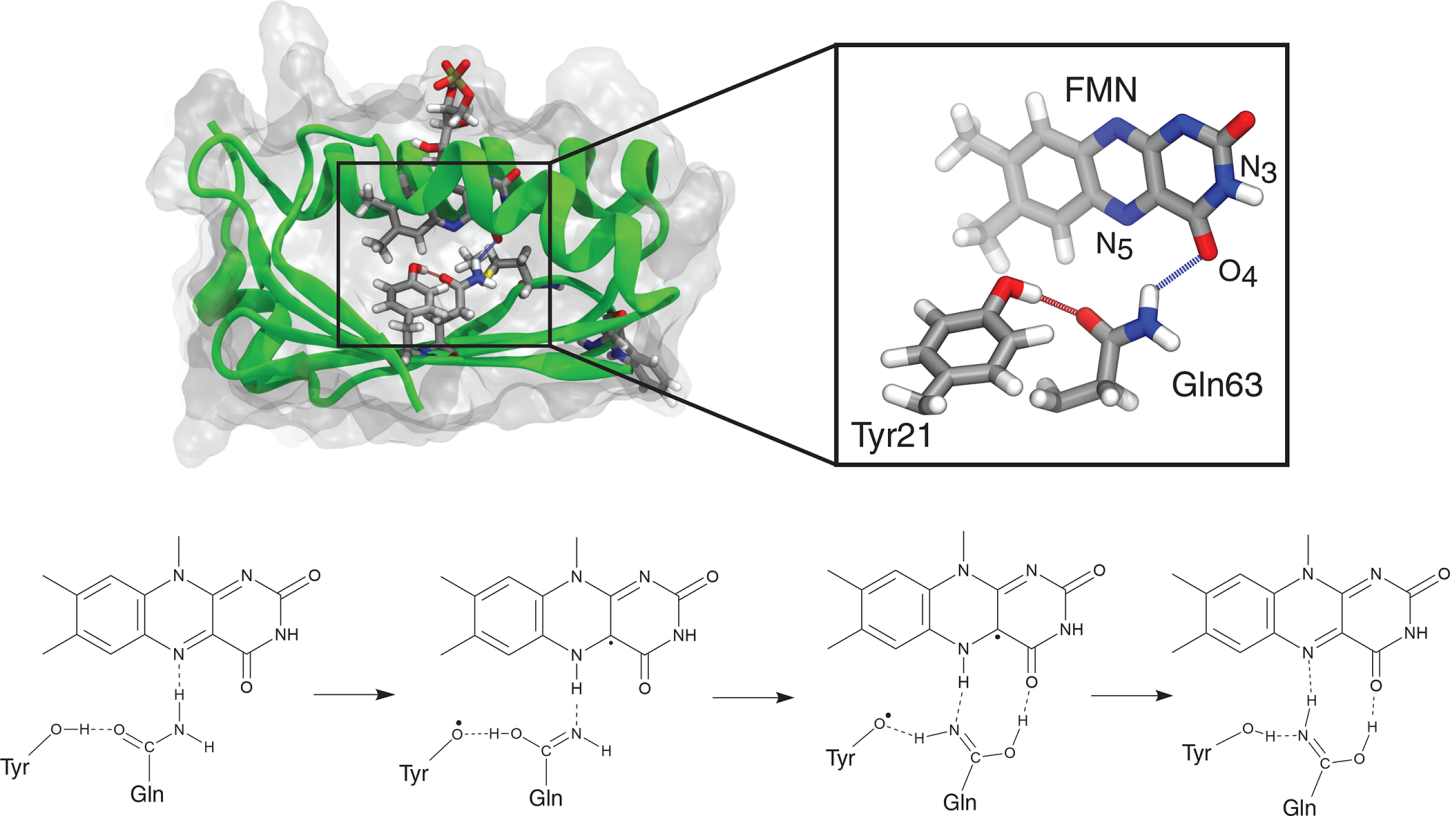
Structure of AppA and proposed mechanism for light state formation. The top panel shows the structure of the Met_*in*_ AppA as obtained in previous work. The inset shows the flavin isoalloxazine ring and the putative residues involved in the photoactivation. The bottom panel shows one of the proposed mechanisms for the photoactivation of the BLUF domains.

Our QM model comprises the isoalloxazine ring of FMN and the hydrogen-bonded Tyr21 and Gln63 residues (Figure 1). The rest of the holoprotein and the solvent were treated with the polarizable AMOEBA force field. We started from 10 uncorrelated structures obtained on a microsecond MD of the Met_*in*_ dark state,^57^ from which GS equilibration QM/AMOEBA MDs were run to generate initial conditions for the excited-state dynamics.

Since the Tamm–Dancoff approximation (TDA) of TDDFT has been successfully used to predict at least the first stage of the photoactivation mechanism of another (Slr1694) BLUF protein,^49^ initially we chose to adopt this QM method to study the same process in AppA. The absorption of blue light that induces the process was simulated by means of a sudden switch from the S_0_ to the S_1_ potential energy surface. Ten TDA/AMOEBA simulations were propagated adiabatically on the S_1_ state, which at first showed characteristics of a locally excited (LE) state on the flavin. For the forward PCET to occur, the CT state should become lower in energy than the LE one, changing the nature of S_1_, which becomes a diradical charge-separated Tyr21^•+^FMN^•–^ state. Following the excited-state dipole moment (Figure S2), we could determine that 1 over 10 trajectories gave the LE → CT transition after ∼2 ps of simulation. Immediately after this transition, we observed the first proton transfer from tyrosine to glutamine, as seen by the proton-donor and proton-acceptor distances in Figure 2.

**Figure 2:**
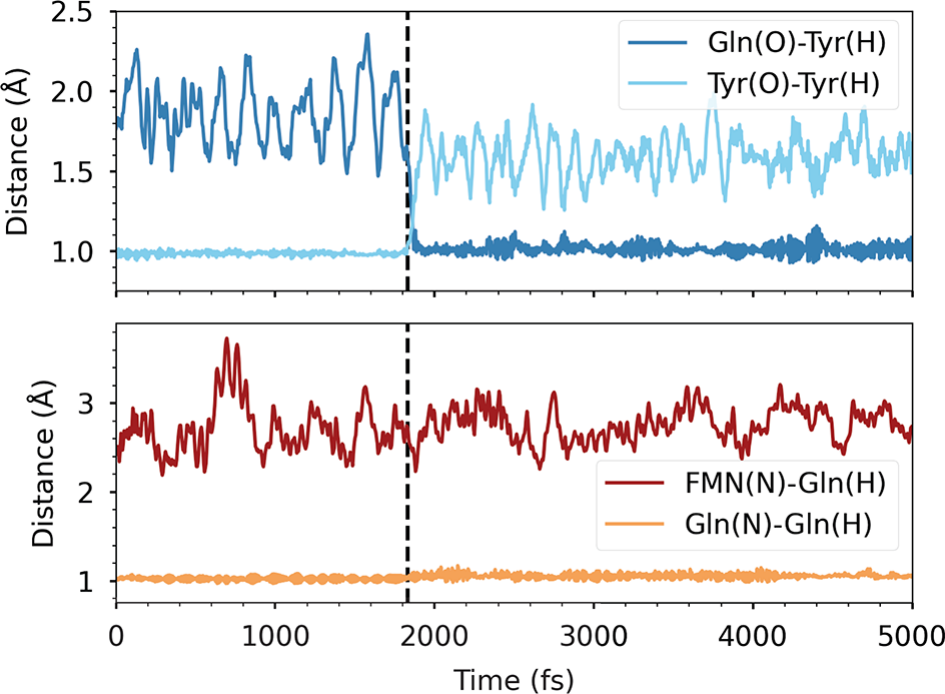
Proton-transfer coordinates for the Tyr-Gln (top) and Gln-FMN (bottom) forward PCETs along the successful TDA/AMOEBA simulation. The black vertical line indicates the time of the charge transfer. Proton-donor distances are depicted with light colors, whereas proton-acceptor distances are dark-colored.

However, no other significant events were detected in the 5 ps simulation. Unexpectedly, the system remained frozen with a positively charged glutamine close to a negatively charged flavin. The simulation was extended to 10 ps in total, without significant changes. In addition, we could observe negative excitation energies, which are a symptom of instability of the closed-shell GS. This was also observed by Goings et al.,^49^ after the first proton transfer, or sometimes after the second proton transfer, and indicates that TDA, from this point on, is not appropriate anymore for describing the dynamics.

Although TDA/AMOEBA proved unable to simulate the entire PCET mechanism, our simulations gave some encouraging results. We found a probability of charge transfer (10%) similar to that obtained by Goings et al. for Slr1694 (9%),^49^ which indicates that the LE → CT transition can indeed occur in the AppA photoreceptor. To simulate the rest of the PCET, Goings et al. changed the QM description along the trajectory to spin-flip TDA/MM. Here, instead, we use a ΔSCF/AMOEBA to propagate the dynamics on the CT potential energy surface by choosing the selected diabatic state at the start of the simulation. To assess the validity of $\Delta$SCF for this kind of systems, we replicated the gas-phase two-dimensional PESs reported in ref (100) (see Figure S1). These calculations showed that, despite its single-reference character, ΔSCF is able to capture the nature of excited states, reproducing qualitatively CASSCF-NEVPT2 PESs. To describe the photoinduced electron transfer, we built the CT state by moving an electron from π_Tyr_ to π_FMN_^*^. Moreover, as ΔSCF employs an unrestricted SCF approach, it is possible to describe diradical species, regardless of the extent of charge separation. Namely, the SCF will find the state of the same nature whether it is the excited or ground state. From now on, these trajectories will be referred to as CT-state MDs.

A proof of the ability of ΔSCF/AMOEBA to follow the selected diabatic state is reported in Figure S5. The plot shows that the values of the ES dipole moment extracted from the dynamics can be associated with a CT state both before and after the intersection between LE and CT. The slight decrease is caused by the first proton transfer, which reduces the charge separation of the system.

Along the MDs, the first proton transfer (PT) occurs on average ∼85 fs after the charge transfer (Table S1), and the second proton transfer occurs ∼125 fs after the first. These time scales indicate a very fast double PT, which, however, is conditional on reaching the CT state. As we observed before with the TDA/AMOEBA simulations, attaining the CT state is a rare event, in that it occurs in ∼1/10 of the trajectories. Therefore, the actual rate of PCET is determined by the time scale for reaching the CT state, while the subsequent PTs are always ultrafast.

After the double PT, the system reaches a neutral diradical GS, where Gln63 is present in its imidic acid tautomer and the flavin is protonated in its semiquinone form (FMNH). As shown in Figure 3a, after ∼1 ps in our representative trajectory, Gln63 starts rotating, and the hydrogen atom that is now bonded to Gln(O) moves away from Tyr21 and forms an H bond with the O4 atom of the flavin ring. (See also the Supporting Information movie). This rotation is possible only because the Gln OH group is oriented upward, in the ZE tautomer. After rotation, Gln63 features two H- bonds with FMNH and one with Tyr21 (top right of Figure 3). However, not all trajectories featured the rotation of Gln63 within the 5 ps (Figure S4). In fact, only in the six trajectories where the ZE tautomer is formed does Gln63 rotate (Figure 4a). Most of the trajectories instead ended up with the EE imidic acid tautomer of Gln63 (Figure 4b), in which the OH hydrogen is oriented opposite to the flavin.

**Figure 3:**
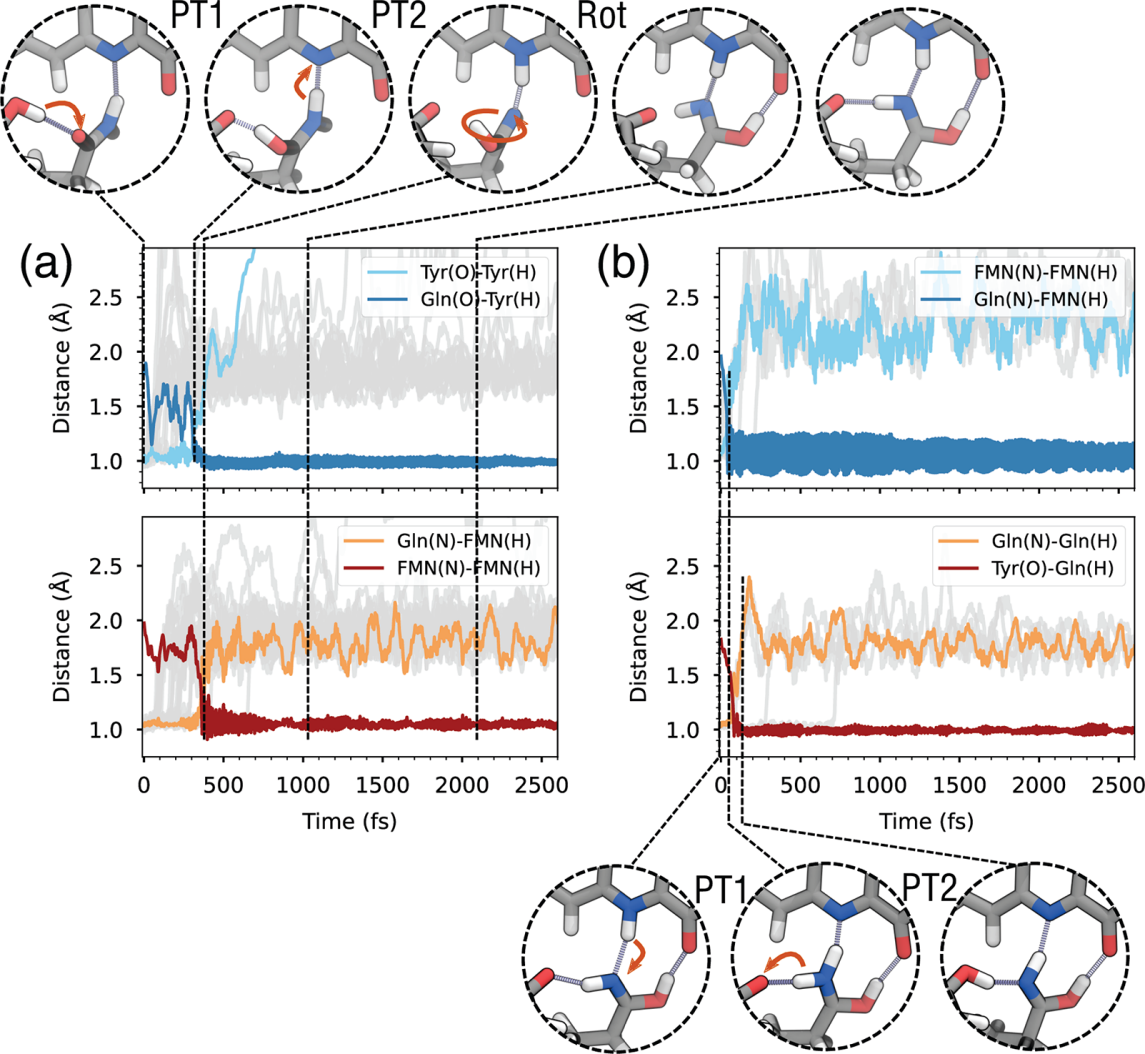
Forward and backward photoinduced PCET in AppA. (a) Proton-transfer coordinates for the Tyr-Gln (top) and Gln-FMN (bottom) forward PCETs along the first 2.5 ps of the CT state dynamics. Black vertical lines indicate the time stamps of the snapshot shown above. (b) Proton-transfer coordinates for the FMN-Gln (top) and Gln-Tyr (bottom) backward PCETs along the ground-state dynamics. Black vertical lines indicate the time stamps for the snapshots below. In all plots, the top/bottom panels show the coordinates involved in PT1/PT2, respectively. Colored lines refer to one representative trajectory, while gray lines represent the remaining trajectories and are shown only for the distance representing the breaking O–H or N–H bond.

**Figure 4:**
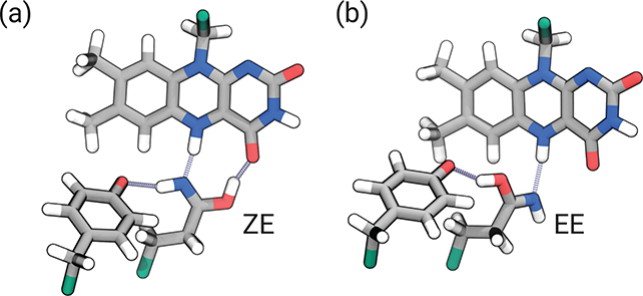
Tautomers of the imidic acid group obtained at the end of CT-state MDs. (a) ZE tautomer (after the rotation). (b) EE tautomer.

In addition to this general behavior, two trajectories formed an EE tautomer after the double PT, switching to the ZE tautomer during the CT-state MD. Since the system requires a few picoseconds to rotate and establish all of the hydrogen bonds, these trajectories did not show a complete rotation in 5 ps. We extended these simulations to 10 ps, finally achieving six trajectories that ended with a rotated imidic acid group.

Starting from the final structure of the CT-state MDs, we simulated a back-electron transfer to the closed-shell state by manually moving an electron back to the Tyr and followed the subsequent dynamics. The closed-shell state features a positively charged protonated flavin and a deprotonated Tyr anion. Initially, this state is higher in energy than the diradical state, owing to charge separation. However, this closed-shell configuration corresponds to the global GS of these structures; therefore, these dynamics will be denoted as GS trajectories. The proton-transfer coordinates during the GS dynamics are shown in Figure 3b for those trajectories that start from the ZE imidic acid tautomer. In five over six trajectories, the FMNH N–H bond breaks, and the hydrogen transfers to the Gln (PT1), followed shortly by PT2 from the opposite hydrogen of the Gln amide group to the Tyr (Figure 3, bottom, Supporting Information movie). In all trajectories, the double PT was completed within 1 ps from the start of the GS MD (Table S2).

The final GS equilibrated structure (bottom right of Figure 3) features a neutral system with protonated Tyr21 and deprotonated FMN, but with Gln63 in its imidic acid ZZ tautomer. The imidic acid is oriented opposite to the initial amide Gln found in the dark structure and is stabilized by H-bond interactions with both FMN’s N5 and O4 atoms and with the hydroxyl group of Tyr21.

Khrenova et al.^47^ investigated two pathways (A and B) for the radical pair recombination, which could occur either from the EE or the ZE tautomeric form of Gln. Our simulations show that, after recombination at the ZE geometry, the reverse PCET yields the tautomeric ZZ form as in pathway A of ref (47) (Figure 5a). To investigate recombination from the EE form, we consider the 14 trajectories where Gln remains in the EE tautomer and does not rotate. Upon charge recombination, these trajectories show a double PCET that completely mirrors the CT-state process in reverse. That is, after back-ET, the system returns to a neutral state featuring the normal amide tautomer of Gln (Figure 5b). This suggests that the rotation of Gln is necessary to move to a different state of the active site, and pathway B of ref (47) does not occur. Conversely, we observed a switch from EE to ZE in two CT-state simulations and the subsequent rotation, reaching the condition for the ZZ tautomer formation after the recombination (Figure 5c). This suggests the possibility that also the other EE tautomers (with enough time) can convert to the ZE form and reach the light-adapted state.

**Figure 5:**
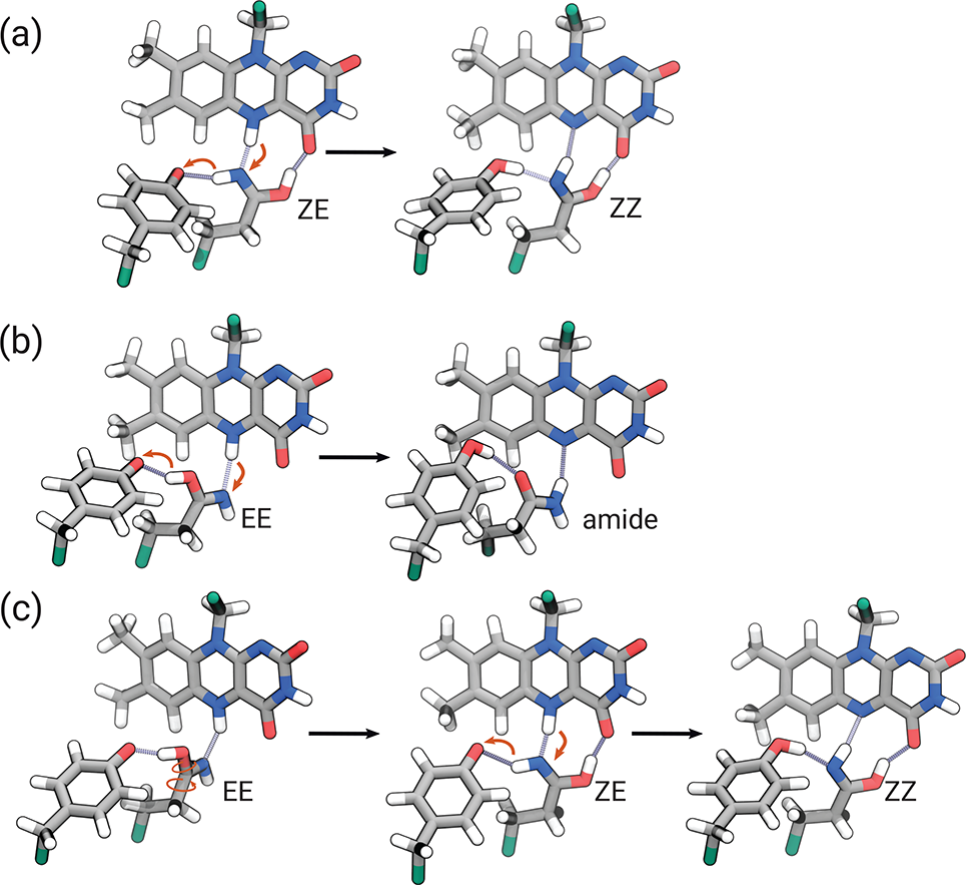
Pathways of the recombination process for the three possible tautomers of the imidic acid group obtained at the end of CT-state MDs. (a) ZE to ZZ tautomer conversion. (b) EE tautomer evolving to the amide form. (c) Switch from EE to ZE tautomer during the CT-state simulation and subsequent formation of the ZZ tautomer.

To conclude, our simulations closely resemble the picture proposed by Domratcheva et al.^45^ and outlined in Figure 1. Importantly, we have not imposed a reaction coordinate for the mechanism or any constraint other than the population of the CT state at the beginning of our simulations. Our results are also analogous to those obtained by Goings et al.^49,50^ on Slr1694_BLUF_. This strongly indicates a conserved mechanism among different BLUF domains, a conclusion that has been doubted in the literature exactly for the supposed uniqueness of AppA for which no spectroscopic signal relative to radical species was directly observed.^54^ However, complex multiexponential kinetics was observed, which is consistent with multiple relaxation pathways.^51^ If charge recombination and backward PCET are much faster than the attainment of the CT state, then the population of radical species will be always small, which would prevent their observation.

This application shows the potentials of the combination of a polarizable MM model with a ΔSCF description in accurately but feasibly simulating excited-state processes of molecules embedded in proteins. In particular, the PCET process investigated here is a perfect application of this strategy as it requires a QM description suited for treating radical species and a model of the environment that can “adapt” to the generation of CT states and their evolution in time.

*Computational Details*. We started from 10 QM/AMOEBA MD simulations presented in a previous work.^57^ Here, we extended these simulations after placing the side chains of Tyr21 and Gln63 in the QM part together with the isoalloxazine ring of FMN. The ribityl tail of FMN, the entire protein, and a 30 Å solvent shell around the chromophore were included in the MM part and treated at the AMOEBA level. In all QM/AMOEBA MDs, the QM part was treated at the ωB97X-D/6-31G(d) level. All residues beyond 22 Å from the flavin were kept frozen, and their AMOEBA polarizabilities were neglected to speed up the calculations. Simulations were propagated in the NVT ensemble using the Bussi thermostat^58^ with a time constant of 0.1 ps and an integration step of 0.5 fs, as done in our previous work.^57,59^ The system was equilibrated classically at room temperature, so no zero-point energy has been included. Each GS QM/AMOEBA MD (from now on denoted with the letters A··· J) was run for 12 ps to allow equilibration and to sample initial conditions. Three initial conditions (positions and velocities) were extracted from the last 10 ps of each trajectory, with 5 ps spacing between them, for a total of 30 initial conditions.

The CT states were described using ΔSCF coupled with AMOEBA.^21^ Initial guesses were obtained by moving one electron from the π_Tyr_ to the π_FMN_^*^ orbital. The STEP method^14^ was employed to converge the SCF on the CT state during ΔSCF/AMOEBA CT-state simulations. The reference orbitals used for the STEP method were taken from the previous step of the simulation, and the Grassmann extrapolation scheme^42^ was used to construct the guess density matrices (α and β) from the previous six simulation steps. Of the 30 dynamics runs, 10 failed because of SCF convergence issues and were discarded. The remaining 20 simulations were run for 5 ps without interruptions. Two MDs, which attained the ZE Gln tautomer only after 4 ps, were extended for an additional 5 ps to observe the rotation of Gln. The final snapshots of CT-state MDs were used as initial conditions for the GS dynamics. In these MDs, the system was propagated using closed-shell SCF to simulate backward PCET. The lowest closed-shell state was initially higher in energy than the diradical state and featured a negative charge on tyrosine while the flavin was positively charged. After the backward PCET, the closed-shell state was always the ground state. The closed-shell MDs were propagated for 5 ps, but no notable change was observed after the backward PCET.

## Supporting Information Available

ΔSCF approaches and their implementation; details of the Grassmann extrapolation for open-shell systems; two-dimensional gas-phase PES scans; additional plots for TDA/AMOEBA simulations; plot of the EE–ZE rotation; LE-CT difference along a CT-state simulation; and times to first and second proton transfers in CT-state and ground-state SCF simulations (PDF)
Movie: representative trajectory for the forward- and backward-PCET mechanisms (MP4)
Transparent Peer Review report available (PDF)
