# Supplementary material for: Fast Method for Excited-State Dynamics in Complex Systems and Its Application to the Photoactivation of a Blue Light Using Flavin Photoreceptor
Source: J Phys Chem Lett. 2023 Jan 30;14(5):1222–9. doi: 10.1021/acs.jpclett.2c03797 (PMC9923743; doi:10.1021/acs.jpclett.2c03797)
Supplement: Supplementary file 1 — jz2c03797_si_001.pdf [file jz2c03797_si_001.pdf]

Supporting Information: A Fast Method for Excited State Dynamics in Complex Systems and its Application to the Photoactivation of a Blue-Light Using Flavin Photoreceptor

Patrizia Mazzeo[1], Shaima Hashem[1], Filippo Lipparini[1], Lorenzo Cupellini[1], and Benedetta Mennucci[1]

[1]Department of Chemistry and Industrial Chemistry, University of Pisa, 56124 Pisa, Italy

S1 ΔSCF approaches and their implementation

In this section, we present with greater detail the two ΔSCF methods we focused on, i.e. initial Maximum Overlap Method (iMOM) and State-Targeted Energy Projection (STEP).

In iMOM[1], reference orbitals are that of the excited guess determinant and new occupied orbitals are selected following a similarity criterion. Let us define the overlap $\boldsymbol{O}$ between current MOs and reference MOs as

$$\boldsymbol{O} = (\boldsymbol{C}^{\text{reference}})^{\dagger} \boldsymbol{S} \boldsymbol{C}^{\text{current}}. \tag{S1}$$

The sum over the row index of the overlap matrix $\boldsymbol{O}$ produces p_j, which indicates the projection of the j-th new orbital onto the space of old occupied orbitals:

$$p_j = \sum_{i}^{n_{\text{occ}}} O_{ij} = \sum_{\nu}^{N_{\text{b}}} \left[\sum_{\mu}^{N_{\text{b}}} \left(\sum_{i}^{n_{\text{occ}}} C_{i\mu}^{\text{reference}} \right) S_{\mu\nu} \right] C_{\nu j}^{\text{current}}. \tag{S2}$$

In eq. S2, i and j are occupied MOs indices and μ and ν refer to atomic orbital basis functions. New occupied orbitals are selected as the orbitals that have the highest values of p_j, which means orbitals that are "similar" to reference orbitals. The advantage of iMOM is that the information contained in the guess, namely the reference determinant that is orthogonal to the ground state and that targets a specific excitation, is retained through the iterative optimization. Therefore, no gradual collapse of the wavefunction onto the ground state is possible.

STEP was presented by Carter-Fenk *et al.*[2] as a robust and efficient alternative to iMOM. This is a completely different approach because it employs the Aufbau principle to choose new occupied

orbitals. However, the constraint which allows the solution to remain in the excited state is a level shift of the virtual orbital energies [2]. Let us consider the operator $\hat{Q}$, which is a projector onto the virtual space:

$$\hat{Q} = \sum_{a}^{n_{\text{virt}}} |\psi_a\rangle \langle\psi_a| . \tag{S3}$$

This projector can be expressed in the atomic orbital basis set as follows:

$$\hat{Q} = \sum_{a}^{n_{\text{virt}}} |\psi_a\rangle \langle\psi_a| = \sum_{\mu\nu}^{N_{\text{b}}} \sum_{a}^{n_{\text{virt}}} |\mu\rangle C_{\mu a} C^{*}_{\nu a} \langle\nu| = \sum_{\mu\nu}^{N_{\text{b}}} |\mu\rangle \boldsymbol{Q} \langle\nu| . \tag{S4}$$

The level shift is performed by adding the matrix form of eq. S4 to the Fock (or Kohn-Sham) matrix, with a parameter η which establishes the amount of level shifting. The new Fock matrix $\boldsymbol{F'}$ is written in eq. S5 and replaces the conventional Fock matrix during the whole iterative procedure.

$$\boldsymbol{F'} = \boldsymbol{F} + \eta \boldsymbol{SQS}. \tag{S5}$$

This expedient can be used to facilitate the convergence to the excited state solution when an excited determinant is given as a guess. Indeed, if the energy of virtual orbitals is increased such that all virtual orbital energies of the excited guess determinant are greater then all the occupied ones, the desired set of occupied orbitals is simply retained with the Aufbau occupation. At the first step, the projector $\hat{Q}$ is chosen on the basis of the reference orbitals, which are usually the same as the ground-state orbitals but with a different occupation targeting an excited state.

Note that the level shift parameter η must be carefully chosen, because a high level shift can reduce excessively the occupied-virtual orbital rotations, resulting in a slower convergence. Conversely, a too small level shift cannot avoid the wavefunction collapse to the ground state. The equation for η suggested by Carter-Fenk *et al.* [2] is

$$\eta = |\epsilon_{\text{HOMO}} - \epsilon_{\text{LUMO}}| + \epsilon' \tag{S6}$$

where ϵ' is an empirical parameter and it is often set to 0.1 Hartree.

S2 Details on the Grassmann extrapolation for open-shell systems

In this section we briefly present the details of the generalization of Grassmann extrapolation [3] to open-shell systems. This allows us to use this procedure for reducing the computational cost of ΔSCF/MM excited-state simulations. The theory behind the extrapolation can be easily extended to unrestricted SCF by working separately on α and β electrons. The new extrapolation problem can be formulated as follows: there are two sets of pairs $(\mathbf{R}_i, D_i^{\alpha})$ and $(\mathbf{R}_i, D_i^{\beta})$ and a new position vector $\mathbf{R}_n$. The gaol is to guess density matrices $D_{\mathbf{R}}^{\alpha}$ and $D_{\mathbf{R}}^{\beta}$ for that geometry.

$$\mathbf{R} \longrightarrow d_{\mathbf{R}} \begin{cases} \longrightarrow \Gamma_{\mathbf{R}}^{\alpha} \to D_{\mathbf{R}}^{\alpha} = \mathrm{Exp}_{D_0^{\alpha}}(\Gamma_{\mathbf{R}}^{\alpha}) \\ \longrightarrow \Gamma_{\mathbf{R}}^{\beta} \to D_{\mathbf{R}}^{\beta} = \mathrm{Exp}_{D_0^{\beta}}(\Gamma_{\mathbf{R}}^{\beta}) \end{cases}$$

In the scheme, $\mathbf{R}$ is the current geometry and $d_{\mathbf{R}}$ are molecular descriptors (we use the Coulomb matrix). As for the closed-shell case, we find the coefficients $c_{\mathbf{R}}$ that provide the best approximation of $d_{\mathbf{R}}$, and we use these coefficients to perform a linear combination of vectors in the tangent space. There are two tangent spaces, identified by the Grassmann Logarithm based on D_0^{α} and D_0^{β}, which are α and β density matrices at the first MD step. $\Gamma_{\mathbf{R}}^{\alpha}$ and $\Gamma_{\mathbf{R}}^{\beta}$ are the results of linear combinations in the tangent spaces. Extrapolated density matrices ($D_{\mathbf{R}}^{\alpha}$ and $D_{\mathbf{R}}^{\beta}$) are recovered by means of Grassmann Exponential mappings.

Ideally, this extrapolation procedure produces converged density matrices at the current step of simulation. Since we perform some approximations, we can state that $D_{\mathbf{R}}^{\alpha}$ and $D_{\mathbf{R}}^{\beta}$ are the best approximation of converged density matrices, and we can use these matrices as a guess for ΔSCF iterations.

S3 Supplementary Figures

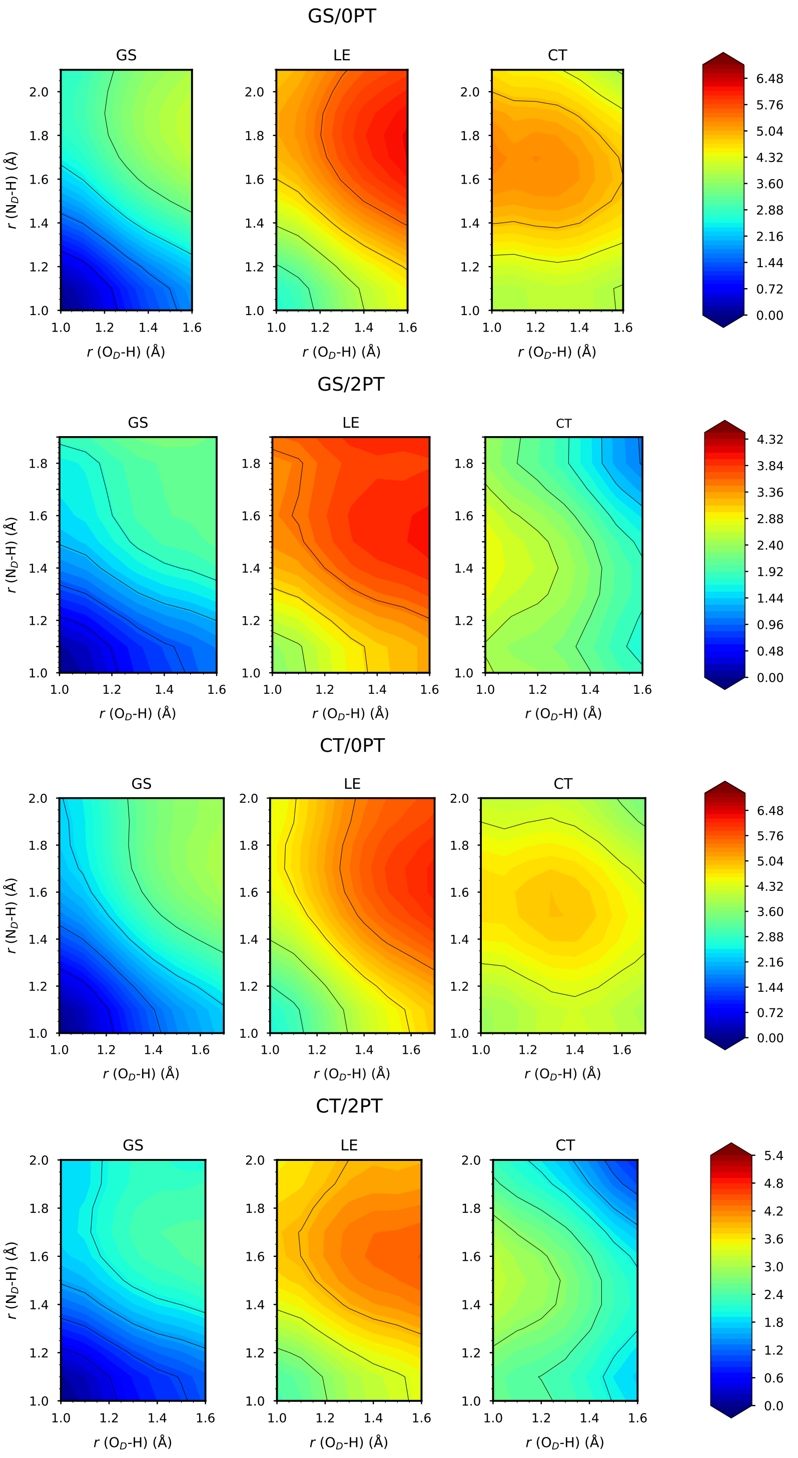

Figure S1: Two-dimensional PESs performed on a rigid scan of the proton transfer coordinates starting from the structures of Ref.[4]. The PESs were computed in gas phase at the (Δ)SCF-ωB97X-D/6-31G(d) level. All energies are given in eV and are calculated relative to the GS before the double proton transfer. The x-axis represents the Tyr-Gln PT coordinate, and the y-axis represents the Gln-FMN PT coordinate.

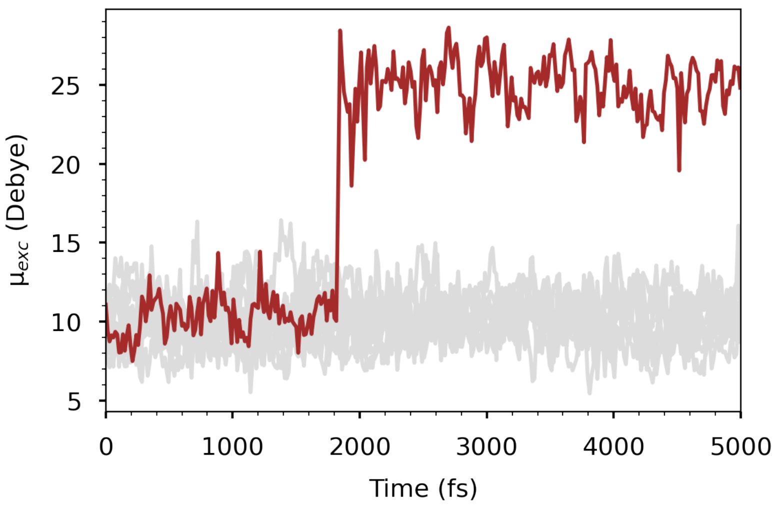

Figure S2: Modulus of the excited-state dipole moment ($\mu_{\rm exc}$) along the successful TDA/AMOEBA simulation. Grey lines in the background reproduce the same plot for all the TDA/AMOEBA trajectories.

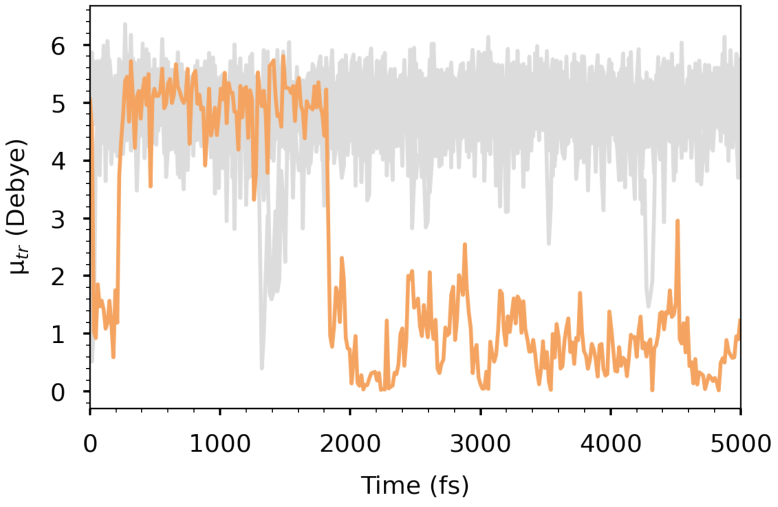

Figure S3: Modulus of the transition dipole moment ($\mu_{\rm tr}$) along the successful TDA/AMOEBA simulation. Grey lines in the background reproduce the same plot for all the TDA/AMOEBA trajectories.

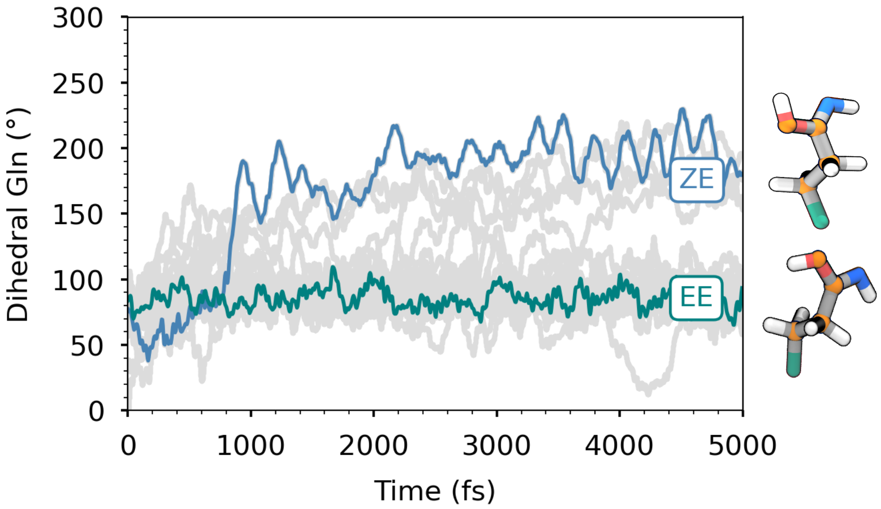

Figure S4: Evolution of the depicted dihedral angle for two representative CT-state MDs. The dihedral involves the atoms depicted in orange in the imidic acid residue, namely the oxygen, the imidic carbon, C_α, and C_β. Grey lines in the background reproduce the same plot for all the CT-state MDs.

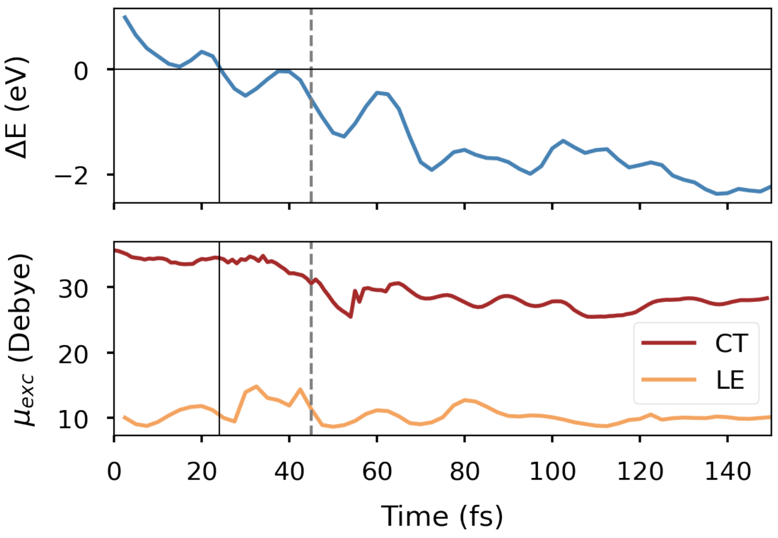

Figure S5: Top panel: $\Delta E = E_{\mathrm{CT}} - E_{\mathrm{LE}}$ along the first 150 fs of a CT-state simulation. E_{CT} values are extracted from the simulation, whereas E_{LE} values are obtained from ΔSCF single-point calculations on the LE determinant, which is constructed by moving an electron from π_{FMN} to π^*_{FMN}. These calculations have been perfomed on 60 geometries extracted from the CT trajectory with 2.5 fs spacing between them. The black vertical line indicates the time of the intersection between LE and CT state and the grey dotted line indicates the time of the first proton transfer. Bottom panel: modulus of the excited-state dipole moment (μ_{exc}) along the first 150 fs of a CT-state simulation. The brown line refers to the values extracted from the dynamics (CT state) and the orange one indicates μ_{exc} values for the LE state at the same geometry.

S4 Supplementary Tables

Table S1: Time stamps for the first and the second proton transfer in the CT-state MDs. The first PT is defined as the time where the Tyr(O)-H bonds becomes longer than the H-Gln(O) bond, whereas the second PT is defined from the Gln(N)-H and FMN(N)-H bonds. The time for the second proton transfer ($\Delta t = t_{\mathrm{second}} - t_{\mathrm{first}}$) is also indicated. The trajectory name refers to the original ground-state sampling trajectory.

Trajectory	t_{first} (fs)	t_{second} (fs)	Δt (fs)
B	44	75	30
D	208	420	212
E	45	655	610
F	113	178	65
G	201	293	92
H	307	360	52
I	56	178	122
J	78	225	147
C'	32	104	72
D'	34	215	181
E'	90	160	70
F'	37	96	59
H'	53	84	31
B''	22	228	206
D''	24	88	64
E''	68	101	33
F''	139	206	67
G''	58	68	10
H''	25	102	77
I''	30	289	259
Mean	83	207	123
St. Dev.	76	145	134

Table S2: Time stamps for the first and the second proton transfer in the ground-state MDs. Only the ground-state MDs that started from the ZE tautomer are reported here. The time for the second proton transfer ($\Delta t = t_{\text{second}} - t_{\text{first}}$) is also indicated. The trajectory name refers to the original ground-state sampling trajectory.

Trajectory	t_{first} (fs)	t_{second} (fs)	Δt (fs)
B	118	717	599
G	216	288	71
H	38	74	36
H'	54	71	17
H''	62	93	31
Mean	98	249	151
St. Dev.	73	277	251

References

[1] Macetti, G. & Genoni, A. Initial maximum overlap method for large systems by the quantum mechanics/extremely localized molecular orbital embedding technique. *J. Chem. Theory Comput.* **17**, 4169–4182 (2021).

[2] Carter-Fenk, K. & Herbert, J. M. State-targeted energy projection: A simple and robust approach to orbital relaxation of non-aufbau self-consistent field solutions. *J. Chem. Theory Comput.* **16**, 5067–5082 (2020).

[3] Polack, E., Dusson, G., Stamm, B. & Lipparini, F. Grassmann Extrapolation of Density Matrices for Born-Oppenheimer Molecular Dynamics. *J. Chem. Theory Comput.* **17**, 6965–6973 (2021).

[4] Sayfutyarova, E. R., Goings, J. J. & Hammes-Schiffer, S. Electron-Coupled Double Proton Transfer in the Slr1694 BLUF Photoreceptor: A Multireference Electronic Structure Study. *J. Phys. Chem. B* **123**, 439–447 (2019).
