# Supplementary material for: Fast Method for Excited-State Dynamics in Complex Systems and Its Application to the Photoactivation of a Blue Light Using Flavin Photoreceptor
Source: J Phys Chem Lett. 2023 Jan 30;14(5):1222–9. doi: 10.1021/acs.jpclett.2c03797 (PMC9923743; doi:10.1021/acs.jpclett.2c03797)
Supplement: Supplementary file 3 — jz2c03797_si_003.pdf [file jz2c03797_si_003.pdf]

jz-2022-037975.R1

Name: Peer Review Information for "A Fast Method for Excited State Dynamics in Complex Systems and its Application to the Photoactivation of a Blue-Light Using Flavin Photoreceptor"

First Round of Reviewer Comments

Reviewer: 1

Comments to the Author

What is the major advance reported in the paper?

This paper uses a QM/MM approach with ΔSCF for the excited state QM region and the AMOEBA polarizable force field for the MM region. This approach is applied to the AppA BLUF photoreceptor and leads to a similar mechanism as was observed previously with a related QM/MM approach for the Slr1694 BLUF photoreceptor.

What is the immediate significance of this advance?

Recognizing the similarities in the forward and reverse PCET reactions, including rotation of the Gln, for the two BLUF photoreceptors is important to the photoreceptor field. The ΔSCF QM/MM approach will also be useful for other applications, leading to broader impact.

Technical suggestions

1. This is a matter of personal style, but the phrase “for the first time” in the abstract is probably not necessary. This is a minor point and is up to the authors.

2. On page 9, the authors state that Goings et al. [49] also observed negative excitation energies after the first proton transfer. From the SI of Ref. 49, it appears that this occurred sometimes after the first but sometimes after the second proton transfer.

3. On page 12, the authors state that they simulated back-electron transfer to the closed-shell state and followed the subsequent dynamics. It would be helpful to explain how they did this. Did they manually transfer the electron back to the tyrosine in a closed-shell configuration and start running GS dynamics? Or did the electron naturally transfer back to the tyrosine in the ΔSCF simulations?

4. For the initial conditions, did the authors include any zero-point energy, as recommended by Mai et al., Front. Chem. 2018, 6, 495? There is debate in the literature about this, and it doesn’t seem like there is consensus, but it might be useful to mention it.

5. The ΔSCF method seems quite useful for this purpose, and it would be interesting to see benchmarking comparing potential energy surfaces or slices to another method, such as CASSCF/PT2 or spin-flip TDDFT. Maybe this was done in previous work for systems similar to this BLUF photoreceptor, and the authors could cite this earlier work.

6. The authors give a nice explanation about how they avoid collapse to the ground state in the ΔSCF method. This approach works well for electronic excitations dominated by the HOMO to HOMO+1 transition, but it might be useful to briefly discuss some of the possible challenges with ΔSCF for excited singlet states, such as spin contamination and triplet instabilities. Did the authors encounter any of these issues in this application, or did everything work easily?

7. The authors mention on page 13 that the radical intermediates might not be observed for AppA because the charge recombination and backward PCET are much faster than CT. Did they find anything different for AppA compared to Slr1694 that would explain why the intermediates could be observed for Slr1694 but not for AppA?

Author's Response to Peer Review Comments:

Reviewer #1

What is the major advance reported in the paper?

This paper uses a QM/MM approach with ΔSCF for the excited state QM region and the AMOEBA polarizable force field for the MM region. This approach is applied to the AppA BLUF photoreceptor and leads to a similar mechanism as was observed previously with a related QM/MM approach for the Slr1694 BLUF photoreceptor.

What is the immediate significance of this advance?

Recognizing the similarities in the forward and reverse PCET reactions, including rotation of the Gln, for the two BLUF photoreceptors is important to the photoreceptor field. The ΔSCF QM/MM approach will also be useful for other applications, leading to broader impact.

Authors' Reply: We sincerely thank the reviewer for the positive comments

Technical suggestions

1. This is a matter of personal style, but the phrase "for the first time" in the abstract is probably not necessary. This is a minor point and is up to the authors.

Authors' Reply: We thank the Reviewer for the suggestion. We have removed the words "for the first time" from the Abstract.

2. On page 9, the authors state that Goings et al. [49] also observed negative excitation energies after the first proton transfer. From the SI of Ref. 49, it appears that this occurred sometimes after the first but sometimes after the second proton transfer.

Authors' Reply: We thank the Reviewer for noticing this imprecision. We corrected as follows:

"This was also observed by Goings *et al.*[49] after the first proton transfer, or sometimes after the second proton transfer, and indicates that TDA, from this point on, is not appropriate anymore for describing the dynamics."

3. On page 12, the authors state that they simulated back-electron transfer to the closed-shell state and followed the subsequent dynamics. It would be helpful to explain how they did this. Did they manually transfer the electron back to the tyrosine in a closed-shell configuration and start running GS dynamics? Or did the electron naturally transfer back to the tyrosine in the ΔSCF simulations?

Authors' Reply: We manually transferred the electron back to the Tyr, in order to create a closed-shell configuration. In order to better clarify this point, we changed the related sentence as follows:

"Starting from the final structure of the CT-state MDs, we simulated a back-electron transfer to the closed-shell state by manually moving an electron back to the Tyr, and followed the subsequent dynamics."

4. For the initial conditions, did the authors include any zero-point energy, as recommended by Mai et al., Front. Chem. 2018, 6, 495? There is debate in the literature about this, and it doesn't seem like there is consensus, but it might be useful to mention it.

Authors' Reply: We have not included an additional zero-point energy, and the system was equilibrated classically at room temperature. We added the following sentence in the Computational Details section:

"The system was equilibrated classically at room temperature, so no zero-point energy has been included."

5. The ΔSCF method seems quite useful for this purpose, and it would be interesting to see benchmarking comparing potential energy surfaces or slices to another method, such as CASSCF/PT2 or spin-flip TDDFT. Maybe this was done in previous work for systems similar to this BLUF photoreceptor, and the authors could cite this earlier work.

Authors' Reply: To the best of our knowledge, the comparison of potential energy surfaces produced with ΔSCF and more accurate methods for complex systems similar to AppA-BLUF is still missing in the literature. However, ΔSCF was used for similar applications before. For instance, in ref. Macetti, G. and Genoni, A. J. Chem. Theory Comput. 2021, 17, 4169−4182, the authors adopted ΔSCF to describe the QM part (the isoalloxazine ring of flavin mononucleotide) of a Flavodoxin protein and compared the ΔSCF spectra obtained with the TD-DFT one. ΔSCF was in a qualitative agreement with TD-DFT.

On the other hand, rigid two-dimensional PES scans of the PT coordinates have been performed for Slr1694-BLUF at the CASSCF/NEVPT2 level in the gas phase [Sayfutyarova et al., J Phys Chem B 2019, 123, 439–447]. We have repeated these PES calculations with ΔSCF, finding a good qualitative agreement with the high-level reference. We have provided these scans in Figure S1 of the Supporting Information. In addition, we added the following sentence to the main text:

"To assess the validity of ΔSCF for this kind of systems, we replicated the gas-phase two-dimensional PESs reported in Ref. 58 (See Figure S1). These calculations showed that, despite its single-reference character, ΔSCF is able to capture the nature of excited states, reproducing qualitatively CASSCF-NEVPT2 PESs."

6. The authors give a nice explanation about how they avoid collapse to the ground state in the ΔSCF method. This approach works well for electronic excitations dominated by the HOMO to HOMO+1 transition, but it might be useful to briefly discuss some of the possible challenges with ΔSCF for excited singlet states, such as spin contamination and triplet instabilities. Did the authors encounter any of these issues in this application, or did everything work easily?

Authors' Reply: We have not encountered triplet instabilities. On the other hand, spin contamination is indeed present, as we directly use the unrestricted-single-determinant energies. It is debated whether it is better to correct for spin contamination or not. However, we note that for CT states the singlet and triplet energies should be very close, and we do not expect the two approaches to give very different results.

As detailed in the Methods section, some of the CT simulations failed because the SCF could not reach convergence. This seemed to occur when there was near degeneracy and mixing between the pi orbitals of FMN and Tyr.

7. The authors mention on page 13 that the radical intermediates might not be observed for AppA because the charge recombination and backward PCET are much faster than CT. Did they find anything different for AppA compared to Slr1694 that would explain why the intermediates could be observed for Slr1694 but not for AppA?

Authors' Reply: We have not found substantial differences between AppA and Slr1694. We compared the times of the first and the second proton transfers (by taking the zero point of time at the charge-transfer) with the results of Goings et al., and they are in good agreement. However, the rate of formation of the charge-transfer state and the subsequent back-electron transfer are the ones determining the observation of intermediates. Our rate of formation of the CT state (~10%) computed with TD-DFT is compatible with the ones of Goings et al., but a much larger statistics would be needed to achieve a quantitative comparison. In addition, the two systems should be simulated with the same MM embedding to obtain a fair comparison.
